# A German translation and adaptation of the knowledge hiding scale

**DOI:** 10.3389/fpsyg.2026.1812789

**Published:** 2026-05-28

**Authors:** Andreas L. Steiner, Victoria K. E. Bart, Martina Rieger

**Affiliations:** Department of Psychology and Sports Medicine, Institute of Psychology, UMIT TIROL – Private University for Health Sciences and Health Technology, Hall in Tirol, Austria

**Keywords:** German translation, knowledge hiding, knowledge hiding scale, psychometric properties, situation-independent knowledge hiding scale, situation-specific knowledge hiding scale

## Abstract

Knowledge hiding, defined as any deliberate act of withholding company-related knowledge from coworkers, has increasingly attracted attention in the scientific literature due to its detrimental impact on organizational performance and dynamics. Despite this growing interest, critical gaps remain regarding the reliable measurement of knowledge hiding across different languages. Accordingly, the aim of this study was to translate, validate, and adapt the knowledge hiding scale by Connelly et al. (2012) for application in German-speaking work environments. To capture different conceptualizations of knowledge hiding, two versions were developed: one situation-specific scale (corresponding to the original scale) and one situation-independent scale. The latter was designed to enable the assessment of knowledge hiding as a relatively stable behavioral tendency, independent of situational factors. Each version was examined in an independent study involving employees from the DACH region (Germany, Austria, and Switzerland; *N* = 480 per study). In Study 1, the original items were translated into German using a back-translation approach; in Study 2, the items were adapted to capture situation-independent behavior. Exploratory and confirmatory factor analysis were performed, replicating the original three-factor structure for both scales. Associations with conceptually related and theoretically opposing constructs provided evidence for both scales as distinct measures of knowledge hiding. Assessing temporal stability via test–retest reliability in Study 2 showed moderate temporal stability, indicating the situation-independent scale’s suitability for measuring knowledge hiding as a relatively stable tendency. Together, the two scales enable a differentiated assessment of knowledge hiding in both situation-specific and situation-independent contexts within German-speaking settings.

## 1 Introduction

Organizations thrive on collaboration. That is, they prosper when their members possess a strong sense of belonging, leading them to openly share ideas, resources, and responsibilities (Edelmann et al., 2020; Mesmer-Magnus and DeChurch, 2009; Spreitzer et al., 2005). In particular, this entails the open sharing of knowledge and information (Wen and Ma, 2021). While it is true that organizations cannot exert direct authority over the intellectual assets of their staff (Connelly et al., 2019), an unhindered flow of information is closely tied to a company’s operations and competitive advantage (Azeem et al., 2021). Yet, despite its crucial role and its frequent perception as a matter of course (Donnelly, 2019), employees frequently refrain from sharing knowledge. Not because of mere indifference, but as a result of deliberate intent and strategy (Siachou et al., 2021). Such practices are commonly referred to as knowledge hiding and are manifested through the purposeful concealment of company-related information from peers (Connelly et al., 2012). Given how knowledge hiding is subtle but prevalent across professional contexts (Semerci, 2019), has adverse effects on organizational performance (e.g., Shen et al., 2025) and the psychological well-being of individual members in organizations (e.g., Xu et al., 2022), systematic scientific investigation of the phenomenon is warranted. Empirical progress, however, hinges on the availability of reliable instruments. Therefore, the present study pursued two distinct research objectives. First, we aimed to translate and validate the situation-specific knowledge hiding scale introduced by Connelly et al. (2012). Second, we modified the scale to develop a situation-independent scale. The latter was done to facilitate the measurement of knowledge hiding as a relatively stable behavioral tendency, independent of situational factors.

## 2 Theoretical background

### 2.1 Knowledge hiding

While often used interchangeably within the broader realm of knowledge-based frameworks (Arain et al., 2024), the absence of knowledge sharing in organizations is not necessarily equivalent to knowledge hiding (Issac and Baral, 2020). Despite their apparent similarity, these constructs differ fundamentally in their underlying motivation (Pereira and Mohiya, 2021). Connelly et al. (2012), who first conceptualized knowledge hiding, defined it as any deliberate act of withholding information requested by other organizational members. In contrast, knowledge sharing comprises the intentional exchange of explicit, that is, structured knowledge, such as procedures, and tacit knowledge, such as experience and know-how (Serenko and Bontis, 2016). As a result, lack of sharing knowledge may be grounded in ignorance or oversight, and is therefore unintentional (Moh’d et al., 2021), while knowledge hiding is always preceded by a deliberate decision to conceal information (Xiao and Cooke, 2019). In addition to stemming from a calculated intention, knowledge hiding requires a specific request by other members of the organization.

Knowledge hoarding is another construct often confused with knowledge hiding (Dash et al., 2023). When hoarding knowledge, one accumulates and retains specific information, but shares it when requested (de Garcia et al., 2022). When hiding knowledge, on the other hand, a knowledge-seeker’s request is deliberately denied (Connelly and Zweig, 2015).

Within the domain of knowledge hiding, Connelly et al. (2012) introduced the most widely accepted differentiation of the construct (e.g., Arain et al., 2024), distinguishing between evasive hiding, playing dumb, and rationalized hiding. Evasive hiding, the most manipulative form of knowledge hiding, typically involves behaviors such as providing misleading information to coworkers or promising to share knowledge in the future without intending to do so (Burmeister et al., 2019). Playing dumb involves knowledge hiders deliberately deceiving others by pretending not to know the requested information or claiming to lack relevant knowledge of the topic (Kang, 2016; Demirkasımoğlu, 2016). Conversely, rationalized hiding entails providing explanations as to why the requested knowledge cannot be shared (Venz and Shoshan, 2021). While evasive hiding and playing dumb involve deception, rationalized hiding does not and is therefore less likely to elicit negative reactions (Offergelt et al., 2019).

Knowledge hiding can be driven by either malicious or prosocial motives (Connelly and Zweig, 2015). Reasons for knowledge hiding may relate to individuals, coworkers, the organization, or the job itself (Anand and Hassan, 2019). Individual reasons may be grounded in feelings of territoriality (Singh and Jha, 2024; Peng, 2013), causing tacit knowledge to be perceived as psychological property (Anand et al., 2020). Further, distrust among coworkers may lead to the denial of requests (Wu et al., 2022). However, coworkers might also conceal knowledge from one another to protect someone (Connelly et al., 2012). Organizational drivers include highly competitive climates (Han et al., 2021) or poor organizational justice (Oubrich et al., 2021). As for job-related triggers, certain role requirements, e.g., protecting sensitive data (Anand and Hassan, 2019), time pressure (Hilliard et al., 2022), and high knowledge complexity (Yuan et al., 2021) can cause knowledge hiding.

Importantly, knowledge hiding results in far-reaching consequences not only for organizations, but also for their individual members (Wen and Ma, 2021). At the organizational level, it is considered to weaken overall organizational performance (Shen et al., 2025), limit innovation capabilities (Duan et al., 2022), impair problem-solving skills (Nauman et al., 2025), and slow down decision-making (Singh and Jha, 2024). Beyond ramifications for organizations as a whole, implications of knowledge hiding extend to individual members by triggering reciprocal behavior (Serenko and Bontis, 2016), undermining trust (Hernaus et al., 2019; Škerlavaj et al., 2023), impacting the productivity and mental health of the affected (Tokyzhanova and Durst, 2026; Xu et al., 2022), and leading to psychological contract breaches (Bari et al., 2020).

### 2.2 Measuring knowledge hiding

As research on knowledge hiding has rapidly expanded over the past decade (Garg et al., 2022), several measures to assess it have been introduced during this period. Gonçalves et al.’s (2023) systematic review of knowledge withholding behavior provides an overview of some of these instruments alongside measures for related constructs. Besides Connelly et al.’s (2012) original scale, this includes shorter scales such as Peng’s (2013), Serenko and Bontis’s (2016), and Rhee and Choi’s (2016) adapted versions. Among these, the scale introduced by Connelly et al. (2012) has emerged as the most popular and widely used to date (Arain et al., 2024), because, unlike its short-form counterparts, it enables capturing the full complexity of the construct (Joo et al., 2025). The scale comprises three subscales (evasive hiding, playing dumb, and rationalized hiding) and consists of 12 items, with four items assigned to each subdimension. In establishing both the construct and the corresponding scale, Connelly et al. (2012) employed a critical incident technique, prompting participants to recall a recent event in which they denied a coworker’s request for knowledge, with explanations provided as to what such a situation might entail. This procedure has since gained broad acceptance and has been employed in several studies to guide participants (e.g., Ain et al., 2023; Pham, 2024; Serenko, 2019; Zhu et al., 2019).

Even though Connelly et al.’s (2012) scale is the most established English-language measure, validated translations remain scarce, leaving a gap in accessibility in other languages. Demirkasımoğlu (2016) translated the original scale and validated it in Turkish to facilitate research on knowledge hiding in academia and Guo et al. (2022) translated it into Chinese. Building on the original scale, Zhai et al. (2023) developed a knowledge hiding scale for online behavior in social networks in English and Chinese. Joo et al. (2025) translated and validated the scale to fit a South Korean context. While these examples illustrate how research has begun to adapt the knowledge hiding scale for use across different language settings, more validated versions are needed (Xia et al., 2022). This necessity is particularly pronounced in German-speaking countries (also referred to as DACH region), where institutions from Germany, Austria, and Switzerland are among the least represented in publications on knowledge hiding (He et al., 2021), even though prior studies including German-speaking samples indicate that knowledge hiding is a relevant phenomenon in this region (e.g., Burmeister et al., 2019; Offergelt and Venz, 2023).

Further, the original knowledge hiding scale relies on a critical incident technique, making it inherently situation-specific (Breunig and Christoffersen, 2016). However, research has also identified personality as a driving force behind knowledge hiding intentions (Arshad and Ismail, 2018; Pan et al., 2018; Demirkasımoğlu, 2016), suggesting that some people tend to hide knowledge more than others (Škerlavaj et al., 2023). For research purposes, it would therefore be desirable to have an instrument that captures knowledge hiding as a stable behavioral tendency, allowing for assessment outside of situational factors.

### 2.3 Aims and objectives

While the above-mentioned studies confirm that knowledge hiding is a relevant workplace behavior in German-speaking contexts, the lack of validated measurement instruments limits further research in this geographical region. Moreover, the situation-specific nature of the original scale restricts its applicability for assessing knowledge hiding as a stable behavioral tendency. To address both issues, we conducted two independent studies. In Study 1, we translated and validated the original, situation-specific scale into German. We expected to replicate the original three-factor structure and demonstrate adequate reliability and validity. In Study 2, we modified the German items to suit situation-independent measurement. We examined whether the adapted scale met the necessary psychometric standards and additionally assessed retest reliability.

## 3 Study 1: situation-specific knowledge hiding scale

Study 1 focused on the German translation and validation of the knowledge hiding scale. After translating the original items from English to German via back-translation, we collected data from employees in the DACH region. We examined the scale’s factorial structure using exploratory and confirmatory factor analyses. In addition, we analyzed internal consistency as well as convergent and discriminant validity.

### 3.1 Methods

#### 3.1.1 Participants

The target group for this study consisted of adult employees living in the DACH region who worked at least 20 h per week and were continuously employed by the same employer for at least 3 months. These criteria ensure that participants are sufficiently exposed to coworker interactions (Venz and Shoshan, 2021). Participants were recruited through non-probability convenience sampling (e.g., Etikan, 2015), i.e., those available at the time of data collection were recruited via a link to the study. The study was approved by the Research Committee for Scientific Ethical Questions (RCSEQ) of the UMIT TIROL University. To determine the appropriate sample size for this study, we chose a ratio of 20 participants per item (20:1) (Gunawan et al., 2021), resulting in a sample size of 240 participants for the 12-item scale. Because we intended to split the sample for exploratory and confirmatory factor analyses (White, 2022), the target sample size was set to 480 participants.

A total of 652 participants completed the survey. After excluding 162 participants who did not meet the inclusion criteria (110 stated that they had never hidden knowledge at work, 62 provided insufficient information), the final sample comprised 480 respondents. Participants were aged between 19 and 63 years (M = 28.7, SD = 7.2). Two hundred seventy-four participants (57.1%) identified as female, 204 (42.5%) as male, 1 as non-binary, and 1 did not wish to say. Average tenure was 3.6 years (SD = 4.99), and average working hours per week were 31 h (SD = 9.72). Four hundred eleven participants were employed either part-time or full-time. Seventeen were apprentices, 3 worked as freelancers, 16 were self-employed, and another 33 selected the “Other” option and entered roles such as working student.

#### 3.1.2 Material

After obtaining permission from the authors of the original scale, we translated the 12 items from English into German. We followed Klotz et al.’s (2023) suggestion and employed a back-translation procedure (Brislin, 1986), supplemented by an iterative approach to ensure the highest possible cultural fit (Behr, 2017; Douglas and Craig, 2007). Items were first translated from English to German and then translated back into English by two external, bilingual individuals. Note that, unlike the English version, in which a binary gender framework was used, all items were adapted to gender-neutral wording to conform with contemporary language norms (e.g., item 1: ‘zugestimmt der fragenden Person zu helfen, ohne dies wirklich vorzuhaben’; original: ‘Agreed to help him/her but never really intended to.’). The 12 items comprised the three subscales evasive hiding, playing dumb, and rationalized hiding, each captured by four items. Although several studies employed a 5-point rating scale to measure knowledge hiding (e.g., Chen et al., 2023; Zhai et al., 2023; Guo et al., 2022; Fatima et al., 2021; Demirkasımoğlu, 2016), we retained the initial 7-point rating scale ranging from 1 (überhaupt nicht; not at all) to 7 (in sehr großem Maße; to a very great extent). Further, similar to the original study, we assessed related constructs. Specifically, scales for knowledge hoarding (4 items) and knowledge sharing (5 items), derived from Connelly et al. (2012), were translated using back-translation as well. Responses were recorded on the same 7-point rating scale as knowledge hiding.

#### 3.1.3 Procedure

Data were collected via an online survey using LimeSurvey.^1^ Participants first accepted the informed consent statement and then provided their demographic data. After that, we employed a critical incident technique and asked participants to describe an incident in which they chose not to share knowledge with a peer. The instructions included a specific description of what this involved (not showing a person how to do something, giving only part of the information needed, or not helping them to learn something). Participants were asked to describe the situation in as much detail as they wished.

After that, participants completed the knowledge hiding scale, referring to the incident they described. Upon completion, the other questionnaires were randomly presented. The average time spent completing the study was 6.3 min.

#### 3.1.4 Data analyses

We randomly split the sample (*N* = 480) into two halves. One half of the sample (*N* = 240) was used to perform an exploratory factor analysis (EFA) to identify underlying factors (e.g., Watkins, 2018). To confirm this factor structure, we used the other half of the sample (*N* = 240) to perform a confirmatory factor analysis (CFA) following the procedure recommended by Swami and Barron (2019). Further, internal reliability, convergent validity, and discriminant validity were assessed.

### 3.2 Results

#### 3.2.1 Exploratory factor analysis

**Figure 1:**
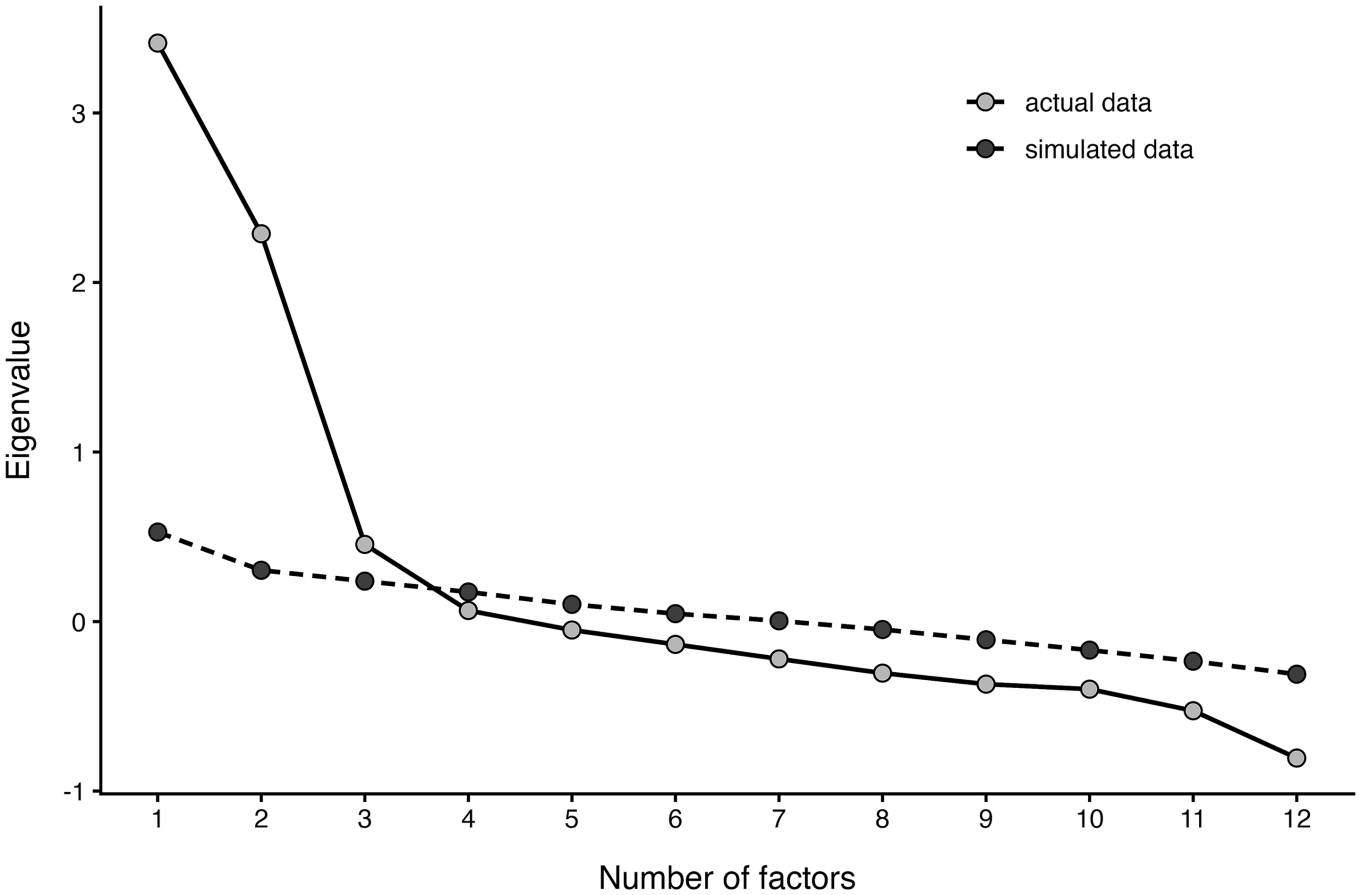
Scree plot of eigenvalues from principal axis factoring with parallel analysis (Study 1).

We performed an EFA, with half of the sample (*N* = 240), using RStudio (Version 2025.09.2 + 418). To ensure the appropriateness of data, we conducted a Bartlett’s test of sphericity and a Kaiser-Meyer-Olkin (KMO) test (Watkins, 2018). Bartlett’s test of sphericity χ^2^(66) = 1,680, *p* < 0.001, indicated that the correlations between the items were adequate to carry out the EFA (Howard, 2016). The KMO test yielded an overall score of 0.79 with all items exceeding 0.60 (lowest = 0.69), again reflecting satisfactory suitability for factor analysis (Kaiser, 1974). We conducted the EFA using principal axis factoring (PAF) with an oblique promax rotation, as we assumed that the underlying factors were correlated (Grieder and Steiner, 2022). To determine the number of factors, we conducted a parallel analysis, comparing eigenvalues from the actual data with those from simulated random data (Horn, 1965), and a scree test (Cattell, 1966). Both methods suggested a three-factor solution, hence aligning with Connelly et al.’s (2012) structure (see Figure 1). The inter-factor correlations were *r* = 0.49 between evasive hiding and playing dumb, *r* = 0.46 between evasive hiding and rationalized hiding, and *r* = 0.11 between playing dumb and rationalized hiding. Notably, these correlations indicate that all subdimensions are related yet capture distinct strategies of knowledge hiding. Overall, the three factors explained 62.5% of variance (rationalized hiding: 26.7%, playing dumb: 22.1%, evasive hiding: 13.7%).

Factor loadings of each item on each of the three factors are depicted in Table 1. Items were deemed satisfactory when they displayed primary loadings above 0.40, showed cross-loadings below 0.30, and differed by at least 0.20 between the primary loading and the highest cross-loading (e.g., Howard, 2016). All but one item fulfilled these criteria. One item (item 3: ‘der fragenden Person gesagt, dass ich ihr später helfen würde, sie aber so lange wie möglich hingehalten.’; original: ‘Told him/her that I would help him/her out later but stalled as much as possible.’) did not meet these requirements and was therefore excluded from further analyses.

**Table 1:** Translated and original items with factor loadings on three factors based on the EFA (Study 1).

Items in German		Items in English	Evasive hiding	Playing dumb	Rationalized hiding
In dieser spezifischen Situation habe ich…		In this specific situation, I…			
1.	zugestimmt der fragenden Person zu helfen, ohne dies wirklich vorzuhaben.	Agreed to help him/her but never really intended to.	**0.47**	0.08	0.06
2.	zugestimmt der fragenden Person zu helfen, habe der Person aber stattdessen andere Informationen gegeben, als sie wollte.	Agreed to help him/her but instead gave him/her information different from what s/he wanted.	**0.96**	−0.17	−0.16
3.	der fragenden Person gesagt, dass ich ihr später helfen würde, sie aber so lange wie möglich hingehalten.	Told him/her that I would help him/her out later but stalled as much as possible.	0.31	0.02	0.25
4.	der fragenden Person andere Informationen angeboten als die, die sie wirklich wollte.	Offered him/her some other information instead of what he/she really wanted.	**0.60**	0.07	0.00
5.	vorgegeben, dass ich die Information nicht habe.	Pretended that I did not know the information.	0.02	**0.83**	−0.22
6.	gesagt, dass ich dies nicht weiß, obwohl ich es wusste.	Said that I did not know, even though I did.	0.06	**0.89**	−0.19
7.	vorgegeben, dass ich nicht wisse, wovon die fragende Person redet.	Pretended I did not know what s/he was talking about.	0.12	**0.65**	0.16
8.	gesagt, dass ich mich mit diesem Thema nicht gut auskenne.	Said that I was not very knowledgeable about the topic.	−0.17	**0.82**	0.14
9.	der fragenden Person erklärt, dass ich es ihr gerne sagen würde, dies aber nicht darf.	Explained that I would like to tell him/her, but was not supposed to.	0.01	−0.10	**0.95**
10.	der fragenden Person erklärt, dass die Information vertraulich ist und nur Personen in einem bestimmten-Projekt zur Verfügung steht.	Explained that the information is confidential and only available to people on a particular project.	−0.08	−0.13	**0.93**
11.	der fragenden Person gesagt, dass meine-Führungskraft nicht erlaubt, dass jemand diese Information weitergibt.	Told him/her that my boss would not let anyone share this knowledge.	0.04	−0.01	**0.89**
12.	der fragenden Person gesagt, dass ich ihre Fragen nicht beantworten werde.	Said that I would not answer his/her questions.	−0.07	0.10	**0.66**

Factor loadings exceeding .40 are depicted in bold.

Internal consistency for the items explored during EFA was evaluated using McDonald’s omega (*ω*) as it does not assume tau-equivalence (Hayes and Coutts, 2020). Following the thresholds recommended in Kalkbrenner (2023), the coefficients for playing dumb (*ω* = 0.87) and rationalized hiding (*ω* = 0.86) demonstrated strong internal reliability, while evasive hiding demonstrated internal reliability slightly below the tentative acceptable guideline of 0.65 (*ω* = 0.63, acceptable value of *ω* = 0.67 before excluding item 3).

#### 3.2.2 Confirmatory factor analysis

**Figure 2:**
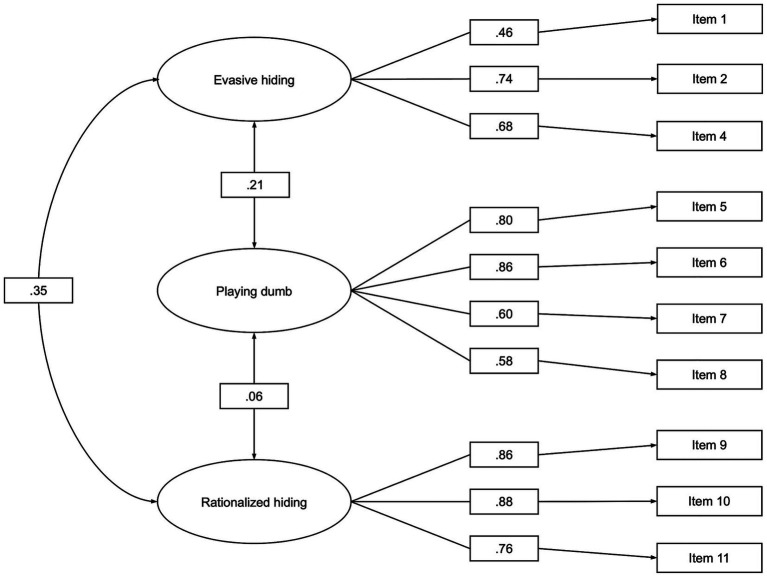
Path diagram illustrating the latent factor covariance and the factor loadings of the 10-item solution on the three factors extracted through confirmatory factor analysis (Study 1).

Using the other half of the sample (*N* = 240), we conducted a confirmatory factor analysis (CFA) to confirm the three-factor structure. We conducted the analysis using the R package lavaan. Figure 2 depicts the standardized factor loadings, all of which were significant and ranged from 0.455 to 0.879. Since one item (item 12: ‘der fragenden Person gesagt, dass ich ihre Fragen nicht beantworten werde.’; original: ‘Said that I would not answer his/her questions.’) showed an insufficient factor loading (*λ* = 0.375), it was excluded from the analysis (Knekta et al., 2019). Subsequently, we reran the analysis using a 10-item solution to improve the model fit. Fit indices were evaluated following commonly recommended cutoffs, with a χ^2^/df ratio below 2 or 3, Root Mean Square Error of Approximation (RMSEA) ranging from 0.06 to 0.08 with confidence interval, Tucker-Lewis Index (TLI) and Comparative Fit Index (CFI) above 0.95, and Standardized Root Mean Square Residual (SRMR) below 0.08 (Schreiber, 2017; Schreiber et al., 2006). The structure consisting of evasive hiding, playing dumb, and rationalized hiding, demonstrated overall acceptable model fit across all indices: χ^2^(32) = 77.13, *p* < 0.001, χ^2^/df = 2.41, CFI = 0.950, TLI = 0.930, SRMR = 0.062, RMSEA = 0.077, 90% CI [0.055, 0.099]. Given the sample-size-sensitive nature of chi-square tests and the large number of participants included in the analyses, it yielding a significant outcome was not interpreted as evidence of model misfit (e.g., Shi et al., 2019). Further, although the TLI was slightly below the recommended cutoff, it was still considered acceptable as other values met the respective criteria (Marsh et al., 2004). In addition, latent factors showed weak to moderate covariance, hence suggesting distinct but partially related constructs.

#### 3.2.3 Internal consistency and construct validity

After conducting EFA and CFA, we tested whether the German scale adequately reflected the underlying construct, again using the second sample from the CFA (N = 240). Results are summarized in Table 2.

**Table 2:** Internal reliability (CR), Convergent (AVE), and discriminant validity (MSV) based on the CFA (Study 1).

Knowledge hiding subscales	CR	AVE	MSV
Evasive hiding	0.67	0.41	0.12
Playing dumb	0.81	0.52	0.05
Rationalized hiding	0.87	0.70	0.12

As a first step, we extended the assessment of internal consistency by complementing the McDonald’s omega coefficients reported in the EFA with composite reliability (CR) estimates. CR was computed for the three knowledge hiding subscales (evasive hiding, playing dumb, rationalized hiding) and deemed satisfactory at values above 0.70 (Cheung et al., 2024). While playing dumb and rationalized hiding exceeded this threshold, evasive hiding did not.

Second, we evaluated construct validity by examining its convergent and discriminant validity. To assess convergent validity, we computed the average variance extracted (i.e., the average amount of variance that a construct explains in its indicators relative to the overall variance of its indicators, AVE) for the three subscales. Values for playing dumb and rationalized hiding fulfilled the required criteria of having AVE values exceeding 0.50 (Hair et al., 2018), while evasive hiding did not. Despite CR and AVE of evasive hiding falling slightly below the recommended thresholds, we decided to retain the construct due to its theoretical relevance and sufficient factor loadings (e.g., Fornell and Larcker, 1981).

Next, discriminant validity was assessed by calculating the maximum shared variance (MSV) for the three knowledge hiding subscales and examining Pearson correlations with related constructs. At the subscale level, discriminant validity was supported, as all three AVE values exceeded their corresponding MSVs (Jiang et al., 2025), indicating that each subscale shares relatively little variance with the others.

Building on this, Pearson correlations were computed among the knowledge hiding subscales, knowledge sharing, and knowledge hoarding. The results are depicted in Table 3. Evasive hiding showed no meaningful associations with either knowledge sharing or hoarding. Playing dumb was negatively correlated with knowledge sharing and positively correlated with knowledge hoarding. In contrast, rationalized hiding was positively associated with knowledge sharing but not with knowledge hoarding.

**Table 3:** Means, standard deviations and Pearson correlations between the three subscales of knowledge hiding (evasive hiding, playing dumb, rationalized hiding) and the scales for other constructs (Study 1).

Construct	M (SD)	Evasive hiding		Playing dumb		Rationalized hiding	
		*r*	*p*	*r*	*p*	*r*	*p*
Knowledge sharing	3.22 (1.47)	0.05	0.492	−0.24	< 0.001	0.11	0.01
Knowledge hoarding	4.78 (1.24)	−0.01	0.944	0.14	0.035	−0.07	0.30

### 3.3 Summary

Findings from Study 1 indicate that the translated German knowledge hiding scale largely reflected the three-factor structure of the original scale and predominantly demonstrated sufficient psychometric properties. Based on the EFA and CFA, 2 items were removed, yielding a 10-item scale. While the two subscales, playing dumb and rationalized hiding, showed robust results throughout all examined criteria, evasive hiding was comparatively weaker as it fell below the recommended threshold for some values. Overall, the results indicate that the scale adequately represents the construct and is able to measure knowledge hiding in a German-speaking sample.

## 4 Study 2: situation-independent knowledge hiding scale

Study 2 aimed to adapt the situation-specific German knowledge hiding scale we developed in Study 1 for use beyond situational contexts. To this end, the translated items were individually rephrased, while new data were collected from employees in the DACH region and again evaluated using exploratory and confirmatory factor analyses. In addition, the new scale’s internal consistency, convergent and discriminant validity, and retest reliability were examined.

### 4.1 Methods

#### 4.1.1 Participants

The same inclusion criteria and sampling approach as in Study 1 were applied in Study 2. Ethical approval for this study was again obtained from the RCSEQ of the UMIT TIROL University. A total of 527 participants completed the survey. Following data screening, 47 participants were excluded due to implausibly short completion times or response patterns indicating insufficient engagement (e.g., straightlining across items), resulting in the final sample again consisting of 480 participants. Age ranged between 19 and 63 years (M = 28.6, SD = 7.5). Three hundred fourteen participants (65.4%) identified as female, 160 (33.3%) as male, 1 as non-binary, and 5 did not wish to say. Participants had an average tenure of 3.3 years (SD = 4.84) and worked an average of 30 h per week (SD = 10). Three hundred seventy were either part-time or full-time employed. Thirty seven were apprentices, 7 were freelancers, 13 were self-employed, and 56 selected the “Other” option and entered roles such as working student.

To assess retest reliability later on, participants were asked whether they would be willing to complete a follow-up survey (see details below). Of the 91 participants who agreed, 47 participated in the follow-up survey. Three participants were excluded from the follow-up data set as they could not be reliably linked to the baseline dataset, leaving 44 participants for the analysis of retest-reliability. The retest sample was aged between 19 and 60 years (*M* = 33.0, *SD* = 10.7). Twenty-eight participants (63.6%) identified as female, 15 (34.1%) as male, and 1 did not wish to say.

#### 4.1.2 Material

To adapt the knowledge hiding scale for applications beyond specific situations and independent of the critical incident technique, we revised the 12 translated items and the instructions. In the instructions, instead of employing a critical incident technique, we asked participants to consider how they typically respond when a team member asks for their knowledge. The items were rephrased to reflect tendencies towards knowledge hiding (e.g., item 8: ‘Wenn ich von einer anderen Person in meiner Organisation um Informationen gebeten werde, sage ich, dass ich mit diesem Thema nicht gut auskenne.’; translation: ‘If someone in my organization asks me for information, I say that I am not very knowledgeable about the topic.’). Responses were collected on a 7-point rating scale (1 = nie; never, 4 = manchmal; sometimes, 7 = sehr oft; very often).

To test the validity of the adapted scale, we again measured several additional constructs. Alongside the knowledge sharing and knowledge hoarding scales already used in Study 1, we measured knowledge disengagement (3 items) (Ford and Staples, 2008) and knowledge contribution loafing (6 items) (Sun et al., 2020; Lin and Huang, 2009), as these constructs have been found to be further constructs associated with knowledge withholding behaviors (Gonçalves et al., 2023). Both scales were translated and adapted from English. Further, we included the German work-related basic need satisfaction scale (18 items) (Grünwald et al., 2024), the German work-related curiosity scale (10 items) (Mussel et al., 2012), and an adapted, that is, self-report rephrased version of the German organizational citizenship behavior scale (20 items) (Staufenbiel and Hartz, 2000). Those scales were selected because previous research indicates that these constructs correlate weakly or negatively with knowledge hiding (e.g., Kurniawanti et al., 2023; Peng et al., 2022; He et al., 2021; Wen and Ma, 2021). Except for the German work-related basic need satisfaction, which is based on a 5-point rating scale, all other scales employed a 7-point rating scale.

#### 4.1.3 Procedure

The procedure was essentially the same as in Study 1, with the following exceptions: Participants were briefed that the study focused on their usual way of dealing with knowledge at work. They were asked to answer the items spontaneously without thinking for too long. After participants completed the adapted version of the knowledge hiding scale, the accompanying questionnaires were randomly presented. The average duration to complete the survey was 11.5 min.

Once participants had answered all items, they were asked whether they would agree to be contacted again after 2 months to take part in a follow-up survey designed to assess retest reliability. If they agreed, they were asked to create and provide an individual code in accordance with specific guidelines, so that data could be stored anonymously but could still be matched to the follow-up survey. In addition, they provided their email so that they could be contacted again (which was stored separately). During the follow-up survey, participants re-entered the code and completed the adapted version of the knowledge hiding scale. Prior to this, participants were also asked whether any changes in their professional circumstances had occurred during the time between the two surveys. If so, they were asked to briefly describe these. A total of 5 participants reported such changes. These participants were retained in the sample, as the reported differences were either minor or unlikely to influence knowledge hiding behavior (e.g., slight reductions in working hours or similar). The average time to fill out the follow-up survey was 6.4 min.

#### 4.1.4 Data analysis

As in Study 1, we randomly split the sample (*N* = 480) into two halves to conduct an EFA and a CFA. Again, internal consistency, convergent validity, and discriminant validity were assessed. Additionally, two-month retest-reliability was computed. For details of the respective analyses, see results section.

### 4.2 Results

#### 4.2.1 Exploratory factor analysis

**Figure 3:**
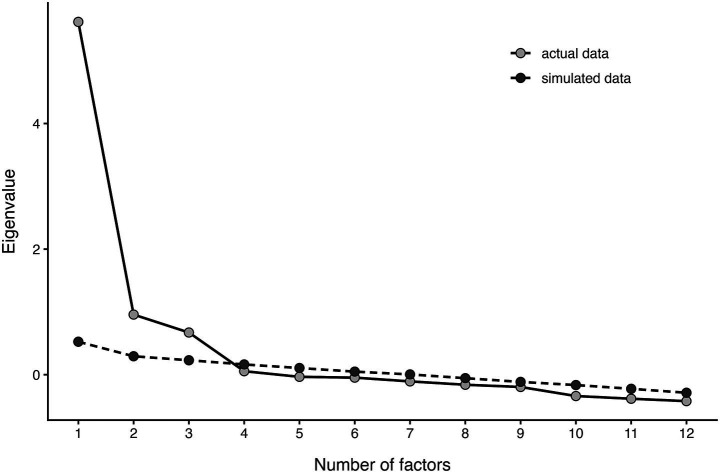
Scree plot of eigenvalues from principal axis factoring with parallel analysis (Study 2).

To perform the EFA, we used one half of the sample (*N* = 240), again using RStudio. Bartlett’s test of sphericity χ^2^(66) = 1939.1, *p* < 0.001, and an overall KMO value of 0.87 with all items exceeding 0.60 (lowest = 0.82), indicated data were adequate to carry out the EFA. Similar to Study 1, we conducted the analysis using PAF and promax rotation. A parallel analysis and a scree test confirmed a three-factor solution (see Figure 3). Inter-factor correlations were *r* = 0.64 between evasive hiding and playing dumb, *r* = 0.47 between evasive hiding and rationalized hiding, and *r* = 0.56 between playing dumb and rationalized hiding. The three factors explained 63.6% of variance (playing dumb: 25.4%, rationalized hiding: 19.2%, evasive hiding: 19.0%). All factor loadings are depicted in Table 4. Items were considered satisfactory based on the same criteria established in Study 1, following Howard (2016). All items fulfilled these criteria. One item (item 12: ‘sage ich der fragenden Person, dass ich ihre Fragen nicht beantworten werde.’; translation: ‘I tell the person asking that I won’t answer their questions.’), however, exhibited a higher loading on a non-target factor than on its intended one and was therefore excluded from further analyses, following Costello and Osborne (2005). As for the internal consistency of the EFA-items, again, playing dumb (*ω* = 0.89) and rationalized hiding (ω = 0.86, ω = 0.81 before excluding item 12) showed strong internal consistency, while evasive hiding demonstrated acceptable internal consistency (ω = 0.79).

**Table 4:** Adapted items with factor loadings on three factors based on the EFA (Study 2).

Adapted Items		Evasive hiding	Playing dumb	Rationalized hiding
Wenn ich von einer anderen Person in meiner Organisation um Informationen gebeten werde…				
1.	stimme ich zu, der fragenden Person zu helfen, ohne dies wirklich vorzuhaben.	**0.58**	0.08	0.08
2.	stimme ich zu, der fragenden Person zu helfen, gebe ihr aber stattdessen andere Informationen, als sie möchte.	**0.87**	0.06	−0.11
3.	sage ich der fragenden Person, dass ich ihr später helfen werde, halte sie aber so lange wie möglich hin.	**0.68**	0.22	−0.10
4.	biete ich der fragenden Person andere Informationen an, als die, die sie wirklich will.	**0.82**	−0.20	0.11
5.	gebe ich vor, dass ich die Information nicht habe.	0.13	**0.77**	0.04
6.	sage ich, dass ich es nicht weiß, obwohl ich es tue.	0.17	**0.79**	−0.01
7.	gebe ich vor, dass ich nicht weiß, wovon die fragende Person redet.	−0.04	**0.92**	0.02
8.	sage ich, dass ich mit diesem Thema nicht gut auskenne.	−0.05	**0.79**	−0.10
9.	erkläre ich der fragenden Person, dass ich es ihr gerne sagen würde, dies aber nicht darf.	0.06	0.11	**0.75**
10.	erkläre ich der fragenden Person, dass die Information vertraulich ist und nur Personen in einem bestimmten Projekt zur Verfügung steht.	−0.02	−0.06	**0.95**
11.	sage ich der fragenden Person, dass meine Führungskraft nicht erlaubt, dass jemand diese Information weitergibt.	−0.03	0.00	**0.86**
12.	sage ich der fragenden Person, dass ich ihre Fragen nicht beantworten werde.	**0.50**	−0.07	0.24

Factor loadings exceeding .40 are depicted in bold.

#### 4.2.2 Confirmatory factor analysis

**Figure 4:**
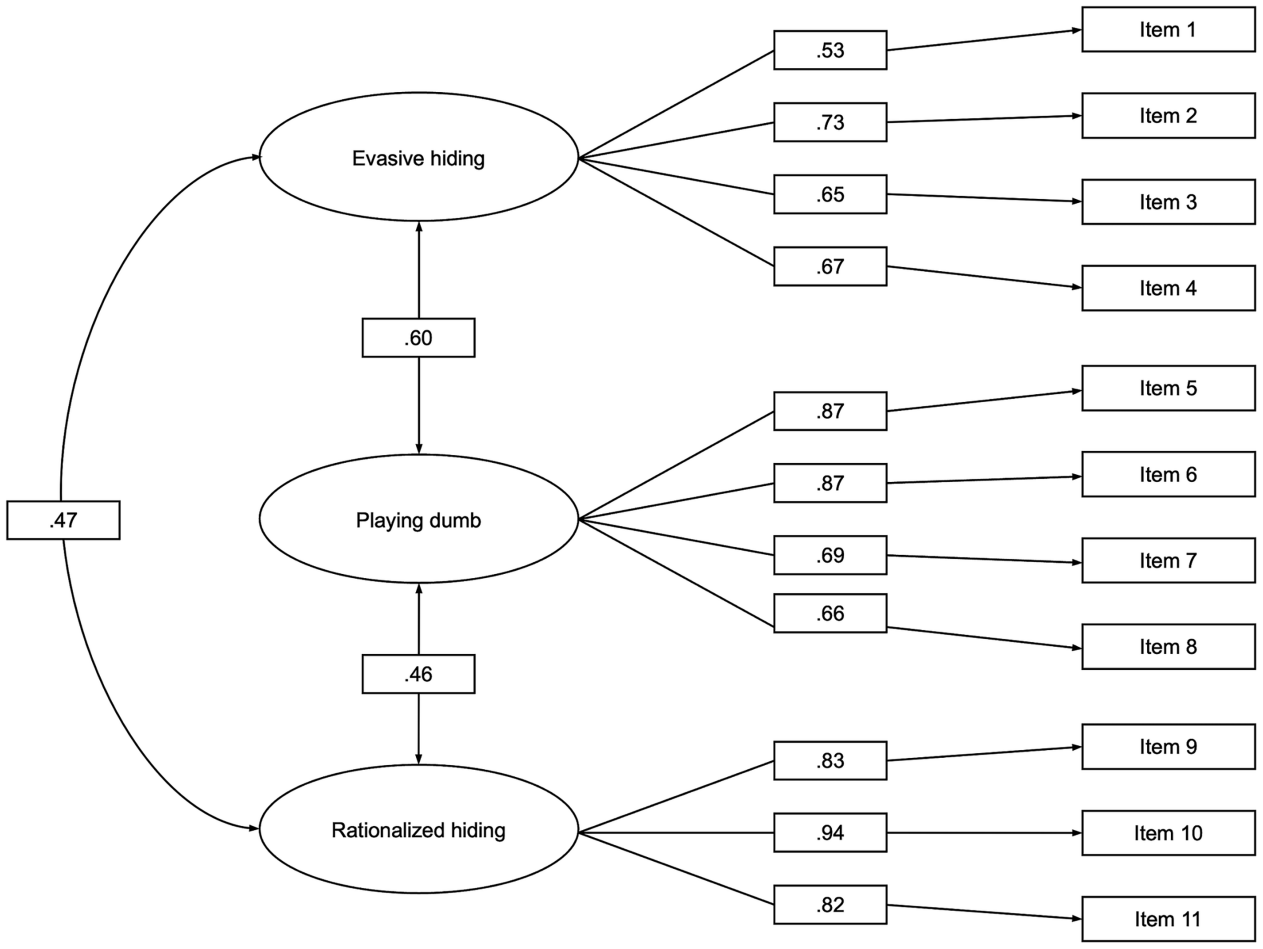
Path diagram illustrating the latent factor covariance and the factor loadings of the 11-item solution on the three factors extracted through confirmatory factor analysis (Study 2).

The other half of the data (*N* = 240) was used to conduct CFA, again using RStudio (lavaan). The analysis yielded excellent model fit across all indices: χ^2^(41) = 53.49, *p* = 0.091, χ^2^/df = 1.30, CFI = 0.99, TLI = 0.99, SRMR = 0.040, RMSEA = 0.036, 90% CI [0.000, 0.060]. Standardized factor loadings are shown in Figure 4. They were significant and ranged from 0.528 to 0.935. Latent factors showed moderate covariance, thereby providing further support for the proposed three-factor model.

#### 4.2.3 Internal consistency and construct validity

Using the CFA sample (*N* = 240), we again extended the EFA’s assessment of internal consistency by computing the CRs of the adapted knowledge hiding subscales and assessing their AVEs to determine their convergent validity. Similarly, we again evaluated discriminant validity by calculating the MSV and Pearson correlations. AVE, CR, and MSV values are depicted in Table 5.

**Table 5:** Internal reliability (CR), Convergent (AVE), and discriminant validity (MSV) based on the CFA (Study 2).

Knowledge hiding subscales	CR	AVE	MSV
Evasive hiding	0.74	0.42	0.35
Playing dumb	0.86	0.61	0.35
Rationalized hiding	0.90	0.75	0.22

Values for evasive hiding, playing dumb, and rationalized hiding exceeded the recommended CR thresholds. Interestingly, while AVE values for rationalized hiding and playing dumb exceeded the 0.50 threshold, thus meeting convergent validity criteria, evasive hiding again fell below the standard. As in Study 1, however, we retained the construct given its theoretical justification (e.g., Fornell and Larcker, 1981). As for discriminant validity, all three AVE values exceeded their respective MSVs.

Additionally, we also calculated Pearson correlations between the three knowledge hiding subscales and the scales we collected alongside (knowledge sharing, knowledge hoarding, knowledge disengagement, knowledge contribution loafing, work-related basic need satisfaction, work-related curiosity, organizational citizenship behavior). The correlations between all scales are shown in Table 6. Evasive hiding and playing dumb showed negative correlations with knowledge sharing while rationalized hiding did not. All three subscales showed small but largely significant positive correlations with knowledge hoarding, knowledge disengagement, and knowledge contribution loafing. For the most part, neither work-related curiosity nor work-related basic need satisfaction (consisting of three subscales) was significantly related with the subscales. Organizational citizenship behavior (consisting of 4 subscales) showed some negative correlations, particularly for the subscales altruism and sportsmanship.

**Table 6:** Means, standard deviations and Pearson correlations between the three subscales of knowledge hiding (evasive hiding, playing dumb, rationalized hiding) and the scales for other constructs (Study 2).

Construct	M (SD)	Evasive hiding		Playing dumb		Rationalized hiding	
		*r*	*p*	*r*	*p*	*r*	*p*
Knowledge sharing	5.66 (0.75)	−0.31	<0.001	−0.33	<0.001	−0.09	0.148
Knowledge hoarding	4.63 (1.16)	0.15	0.023	0.15	0.017	0.19	0.004
Knowledge disengagement	3.20 (1.31)	0.29	<0.001	0.27	<0.001	0.13	0.053
Knowledge contribution loafing	2.67 (1.11)	0.26	<0.001	0.38	<0.001	0.22	<0.001
Work-related curiosity	5.48 (0.94)	−0.11	0.080	−0.04	0.513	0.08	0.246
Work-related basic need satisfaction							
Need for autonomy	3.50 (0.61)	−0.10	0.111	−0.14	0.027	−0.04	0.499
Need for competence	3.91 (0.76)	−0.05	0.430	−0.05	0.478	0.05	0.426
Need for relatedness	3.43 (0.83)	−0.09	0.180	−0.14	0.035	−0.02	0.733
Organizational citizenship behavior							
Altruism	5.48 (1.05)	−0.31	< 0.001	−0.33	<0.001	−0.20	0.002
Conscientiousness	5.68 (0.97)	−0.10	0.107	−0.16	0.017	−0.06	0.396
Sportsmanship	5.32 (0.67)	−0.18	0.005	−0.23	<0.001	−0.04	0.557
Civic virtue	4.88 (1.17)	0.04	0.588	0.01	0.912	0.06	0.353

#### 4.2.4 Test–retest reliability

To assess the temporal stability of the three adapted knowledge hiding subdimensions, we computed intraclass correlation coefficients (ICCs), based on data from *N* = 44 participants, using a two-way mixed effects approach with absolute agreement and average measures in RStudio. Results indicated moderate retest reliability for all three subscales: evasive hiding (ICC = 0.53), rationalized hiding (ICC = 0.55), and playing dumb (ICC = 0.64), supporting the assumption of sufficient stability across two time points (e.g., Koo and Li, 2015).

### 4.3 Summary

Study 2 confirmed a three-factor model for the situation-independent German knowledge hiding scale and again demonstrated adequate psychometric properties for all three subscales. After conducting the EFA, one item was removed, yielding an 11-item solution for this scale. For the most part, the scale demonstrated robust results across all indices, thus providing evidence that it is suitable for assessing knowledge hiding outside situational contexts in a German-speaking sample.

## 5 Discussion

The present paper first aimed to translate and validate a German version of Connelly et al.’s (2012) knowledge hiding situation-specific scale (Study 1) and, second, aimed to adapt it for situation-independent use (Study 2). In both studies, a three-factor structure consistent with the original English scale was obtained. Both scales showed sufficient internal consistency and validity. Further, the situation-independent scale (Study 2) showed sufficient retest reliability.

### 5.1 Situation-specific scale

The initial EFA conducted in Study 1, yielded a three-factor structure consistent with the original English scale (e.g., Connelly et al., 2012), with all but one item (item 3) showing satisfactory loadings (>0.40). These results suggest that, overall, the translated items adequately represent the construct of knowledge hiding and its three subdimensions (evasive hiding, playing dumb, rationalized hiding).

Regarding item 3, several factors might have contributed to it showing insufficient loadings. First, given that this item exceeded thresholds in other studies (e.g., Joo et al., 2025), a likely explanation might be that the German translation did not fully reflect the term ‘stalled as much as possible’. Apart from this, German speakers tend to prefer a more explicit communication style (House, 2006), which may make strategies of initially agreeing to help but deliberately delaying it less typical or prone to being misunderstood. As for the subsequent CFA, one item (item 12: ‘sage ich der fragenden Person, dass ich ihre Fragen nicht beantworten werde.’; translation: ‘I tell the person asking that I won’t answer their questions.’) was excluded due to low factor loadings as well. A possible explanation might lie in the wording or translation of the item, as it is conceivable that a statement indicating not to answer someone’s question might not be interpreted as a form of rationalized hiding. Secondly, conducting a CFA yielded an acceptable model fit. As knowledge hiding is a socially rather undesirable and therefore underreported phenomenon (Demirkasımoğlu, 2016), and data were based on participants’ self-reported workplace experiences, response patterns may have been skewed toward lower scores. This, in turn, might be reflected in this study yielding an acceptable model fit, while Study 2 demonstrated an excellent fit.

For the most part, the translated scale demonstrated satisfactory psychometric properties. Evidence for discriminant validity was found both among the three subscales and in correlations with related constructs, suggesting that the German situation-specific knowledge hiding scale measures a distinct construct. As for internal consistency and convergent validity, two out of three subscales (playing dumb and rationalized hiding) showed sufficient values for CR (>0.70), McDonald’s omega (>0.65), and AVE (>0.50). The third subscale (evasive hiding) showed slightly lower reliability estimates and an AVE value marginally falling below the recommended threshold. However, a closer examination of prior research suggests that such deviations should not be interpreted as definitive evidence of insufficient reliability or validity, as there are also examples that specify less stringent evaluation standards for McDonald’s omega (e.g., Karakaya et al., 2025) or question strict adherence to CR and AVE benchmarks (e.g., Rönkkö and Cho, 2022; Maruf et al., 2021). Finally, the pattern of Pearson correlations across two conceptually related constructs (knowledge sharing and hoarding) provided further support for the scale’s validity in measuring knowledge hiding as intended.

### 5.2 Situation-independent scale

In Study 2, we adapted the knowledge hiding scale for situation-independent use, because some people may tend to hide knowledge more than others (e.g., Škerlavaj et al., 2023). It is therefore desirable to have an instrument that captures knowledge hiding as a stable behavioral tendency, allowing the assessment of the tendency to hide knowledge independent of a specific situation.

The EFA showed issues with item 12 (as was the case in the CFA of Study 1). The item loaded onto the wrong factor, thus supporting the assumption of it being misunderstood. After excluding it from further analysis, performing a CFA using an 11-item scale yielded an excellent model fit. One explanation why the data fit the model better in Study 2 than in Study 1 may be that, unlike in the situation-specific scale, we did not employ a critical incident technique for the situation-independent scale. Given how this method has been associated with social desirability and confirmation bias (Coetzer et al., 2012), omitting it for Study 2 may have led to more consistent and candid responses.

Overall, the situation-independent scale showed satisfactory psychometric properties as well. Internal consistency was satisfactory for all three subscales across all indices. Concerning convergent validity, playing dumb and rationalized hiding met the recommended threshold for AVE, while it fell slightly below for evasive hiding. Although the overall results support the assumption that items were interpreted as intended, the AVE for evasive hiding fell slightly below the conventional reference value in both studies, which may reflect minor translation-related differences in item meaning. Pearson correlations with seven other scales, some conceptually related, others representing theoretically distinct constructs, again supported the scale’s reliable measurement of knowledge hiding. Concerning retest-reliability, intraclass correlation coefficients showed moderate temporal stability, indicating that the adapted scale may, indeed, measure tendencies towards knowledge hiding, rather than context-specific behavior.

### 5.3 Limitations and perspectives

The present study is limited by the non-probability sampling method employed in both studies. Including only participants who were available at the time of data collection, may limit the applicability of the scales to the entire population (Ahmed, 2024). A further limitation, inherent to the nature of the construct, is that the knowledge hiding scale relies on self-reports. Potential distortions in data may therefore arise due to social desirability (Long et al., 2024) or a reference bias (Lira et al., 2022).

Translating the situations-specific knowledge hiding scale into German facilitates future research on knowledge hiding in German-speaking contexts. In addition, given that many studies investigating knowledge hiding call for more cross-cultural research (e.g., Wu and Liu, 2025; Xiao, 2024), providing a validated German translation may contribute to such endeavors. Moreover, providing a situation-independent scale allows the investigation of knowledge hiding as a tendency beyond specific situations. That said, future research may further investigate the stability of knowledge hiding behavior. For instance, it could be that the tendency to hide knowledge is relatively stable as long as one remains in the same workplace. However, knowledge hiding behavior may change in a different working environment. Establishing this through longitudinal studies that assess knowledge hiding over extended periods and across various professions, including people who remain in the same workplace or change jobs, would help to shed light on this issue.

Apart from its academic contributions, the present research offers several important implications for practice. Considering the far-reaching detrimental effects of knowledge hiding on individuals (Kurniawanti et al., 2023) and entire organizations (Chatterjee et al., 2021), both scales provide useful tools to reliably detect and analyze such behavior among German-speaking employees. It may help decision-makers and professionals (such as HRM, management, consultants) to systematically assess whether and to what extent knowledge hiding may occur or has occurred in an organization. Enabling stakeholders to identify knowledge hiding as a prevalent issue could then inform tailored trust- and collaboration-enhancing interventions before detrimental patterns manifest. This may be particularly crucial in contexts known to influence knowledge hiding, such as organizational change (Nguyen et al., 2022), promotions (Guo et al., 2024), or new hires (Rao et al., 2021), where new teams are formed, and when knowledge sharing is essential. Repeated measurements would then allow assessing the effectiveness of such interventions or serve as warning signs of a deteriorating collaborative climate (Hernaus et al., 2019).

## 6 Conclusion

To the best of our knowledge, this paper presents the first validated German translation of Connelly et al.’s (2012) knowledge hiding scale (situation-specific scale, 10 items), and an adaptation to assess knowledge hiding as a behavioral tendency (situation-independent scale, 11 items). This addresses the lack of validated instruments for investigating knowledge hiding in German-speaking countries. Both scales demonstrated adequate psychometric properties. Together, these scales aim to facilitate research on knowledge hiding within German-speaking contexts and provide instruments for practitioners to assess knowledge hiding within organizations.

## Data Availability

The datasets presented in this study can be found in online repositories. The names of the repository/repositories and accession number(s) can be found at: https://osf.io/jn2w5/.

## Footnotes

1
https://www.limesurvey.org/de
